# An unregistered TARDBP mutation in a case presenting with young-onset dementia

**DOI:** 10.1192/bjo.2021.338

**Published:** 2021-06-18

**Authors:** Mahmoud Gad, Walid Nasr, Tareq Qassem

**Affiliations:** 1 Al-Amal Hospital, Ministry of Health and Prevention; 2 Maudsley Health, Al-Amal Hospital, Ministry of Health and Prevention, Mohammed Bin Rashid University Of Medicine and Health Sciences, Institute of Psychiatry, Ain Shams University

## Abstract

**Objective:**

This poster aims to report an unregistered mutation in Transactive Response DNA Binding Protein (TARDBP) gene in a patient presenting young-onset dementia.

Hypothesis: Novel heterozygous mutation in the TARDBP gene is linked to a case of with young-onset dementia.

**Background:**

Pathogenic variants in TARDBP cause autosomal dominant fronto-temporal degeneration, characterized by TDP43-positive inclusions, dystonia, dyslexia, receptive dysphasia, and paraphrasic errors. In addition to the neurocognitive deficits, patients might suffer from cardiomyopathy and amyotrophic lateral sclerosis.

**Case report:**

Molecular genetic analysis of whole-exome sequencing (WES) was carried out for a 45-year-old male patient presenting with cognitive decline and behavioural symptoms.

**Discussion:**

WES Identified the heterozygous variant c.527A > T p.(Lys 176lle) in TARDBP gene. To the best of our knowledge the variant has not been described in the literature so far (HGMD 2019.3). No allele frequencies in the general population have been documented.

**Conclusion:**

We believe that we have identified a novel mutation in the TARDBP gene. This mutation is likely to be linked to this patient presenting with young-onset dementia.

